# Confirmatory Factor Analysis and Differential Relationships of the Two Subdomains of Negative Symptoms in Chronically Ill Psychotic Patients

**DOI:** 10.1371/journal.pone.0149785

**Published:** 2016-02-19

**Authors:** Annemarie P. M. Stiekema, Edith J. Liemburg, Lisette van der Meer, Stynke Castelein, Roy Stewart, Jaap van Weeghel, André Aleman, Richard Bruggeman

**Affiliations:** 1 Department of Rehabilitation, Lentis Center for Mental Health Care, Zuidlaren, the Netherlands; 2 Rob Giel Research Center, University of Groningen, University Medical Center Groningen, Groningen, the Netherlands; 3 Department of Neuroscience, University of Groningen, University Medical Center Groningen, Groningen, the Netherlands; 4 Research Department, Lentis Center for Mental Health Care, Groningen, the Netherlands; 5 Department of Health Sciences, Community and Occupational Medicine, University of Groningen, University Medical Center Groningen, Groningen, the Netherlands; 6 Parnassia Group, Dijk en Duin Mental Health Center, Castricum, the Netherlands; 7 Tilburg University, Tilburg School of Social and Behavioral Sciences, Tranzo Scientific center for Care and Welfare, Tilburg, the Netherlands; 8 Phrenos, Center of Expertise on severe mental illness, Utrecht, the Netherlands; 9 University Center of Psychiatry, University of Groningen, University Medical Center Groningen, Groningen, the Netherlands; Katholieke Universiteit Leuven, BELGIUM

## Abstract

Research suggests a two factor structure for negative symptoms in patients with psychotic disorders: social amotivation (SA) and expressive deficits (ED). Applying this two-factor structure in clinical settings may provide valuable information with regard to outcomes and to target treatments. We aimed to investigate 1) whether the factor structure is also supported in chronically ill patients with a psychotic disorder and 2) what the relationship is between these factors and functioning (overall functioning and living situation), depressive symptoms and quality of life. 1157 Patients with a psychotic disorder and a duration of illness of 5 years or more were included in the analysis (data selected from the Pharmacotherapy Monitoring Outcome Survey; PHAMOUS). A confirmatory factor analysis was performed using items of the Positive and Negative Syndrome Scale that were previously identified to reflect negative symptoms (N1-4, N6, G5, G7, G13, G16). Subsequently, regression analysis was performed on outcomes. The results confirmed the distinction between SA (N2, N4, G16) and ED (N1, N3, N6, G5, G7, G13) in chronically ill patients. Both factors were related to worse overall functioning as measured with the Health of the Nation Outcome Scales, ED was uniquely associated with residential living status. Higher scores for SA were associated with more depressive symptoms and worse quality of life. Thus, SA is most strongly related to level of social-emotional functioning, while ED are more related to living situation and thereby are indicative of level of everyday functioning. This subdivision may be useful for research purposes and be a valuable additional tool in clinical practice and treatment development.

## Introduction

Negative symptoms, such as flattened affect, social withdrawal, apathy and avolition, are core symptoms of psychotic disorders, most notably schizophrenia. At least half of the patients with schizophrenia suffers from negative symptoms [1], which are often already present in the prodromal phase [2] and are relatively stable across the course of illness [3]. Negative symptoms have an invalidating impact on patients’ functioning [4–6] and are associated with lower quality of life [7]. Despite the increased focus on negative symptoms as a subject of research, there is still a paucity of (psychosocial) interventions effective in reducing them. Many patients are left with negative symptoms after their positive symptoms have been partially or completely managed by antipsychotic medication [8]. The lack of substantial improvement in everyday functioning after antipsychotic treatment may therefore be impeded by enduring negative symptoms [9].

An accumulating body of research suggests that negative symptoms are multidimensional [10]. Factor analytic studies across different instruments consistently cause two factors to emerge, namely social amotivation (SA) and expressive deficits (ED) [11–14]. The SA subdomain encompasses social and emotional withdrawal and speaks to involvement with the environment [15]. It refects a reduction of interest in social interactions and life events, and a reduction of self-initiated or maintained behaviors with regard to social events. SA has been linked to deficits in anticipatory pleasure (i.e. failure to signal the salience of positive events), thereby losing the drive to engage in (social) situations and activities [15–17]. Thus, SA can be interpreted as a ‘loss of interest’ [13]. The ED subdomain involves directly observable components such as diminished facial expression, poverty of speech and blunted affect [10,15]. ED is a reduction of verbal and non-verbal emotional responsiveness, reflected by a reduction of communicative expression. ED has been associated with impaired neurocognition [11,15,18] and may reflect a ‘loss of initiative’ [13].

Both factors seem to affect functional and psychosocial outcomes differently [10,19–21]. This has important implications, because many (treatment) studies use total negative symptoms scores, which could average out relationships that are mainly driven by one of the subdomains. That is, when subjects demonstrate different scores on each subdomain (high on SA and low on ED or vice versa), their total of negative symptoms may be similar, while their relationship with outcomes could be different as this may be driven by one factor. Therefore, a distinction in subdomains, and more importantly the understanding of possible differential correlates of these factors, could be of importance for clinical diagnosis, therapeutic decision-making and research on treatment development [10,11]. Literature suggests that SA is most strongly associated with functional outcomes such as employment, number of hospitalizations, instrumental role performance and family functioning [10,14,22,23] and that males may score higher on SA than ED [14]. However, the role of ED is less clear. Components of ED such as blunted or flat affect have been associated with poor social functioning and quality of life as well [24,25], but ED shows weaker associations with outcomes than SA [14] or has no additional predictive value after controlling for SA [16]. Therefore, SA is often seen as the key contributor to the relationship between negative symptoms and functional outcomes [20]. The majority of studies investigating the correlates of both domains have focused on functional outcomes and less on other aspects, such as depressive symptoms, which are common in psychotic disorder [26], and quality life. Investigating whether the subdomains differentially relate to quality of life, can guide treatment strategies to more specific targets. For depressive symptoms, a differential relationship of the subdomains could clarify the inconsistencies with regard to the association between global negative symptoms and depressive symptoms in the literature. However, correlational analysis in one study showed no relationship of either factor with depressive symptoms, but did show an association between SA and quality of life [13].

The two factors have been mostly established in samples of patients with recent onset psychosis [10,11,13], but one study established the factor structure in patients with chronic psychosis and a longer duration of illness [22]. As a consequence, little is known about this factor structure in patients with a psychotic disorder with a longer duration of illness. Considering the paucity of studies investigating the subdomains in chronic populations, replication of the factor structure in this population is needed. And, if the factor structure is replicated, the relationship between these factors and functional outcomes and psychosocial well-being should be examined. Therefore, we aimed to investigate 1) whether the factor structure of negative symptoms can be replicated in chronically ill patients with a psychotic disorder and 2) the relationship between these factors and functioning (overall functioning and living situation), depressive symptoms, and quality of life.

## Methods

### Participants

Data were selected from the Pharmacotherapy Monitoring Outcome Survey (PHAMOUS). PHAMOUS is an annual screening of mental and physical health of patients using antipsychotics and receiving mental health care in the North of the Netherlands. We included all patients between 2011 and 2013, diagnosed with a psychotic disorder, with a duration of illness of more than 5 years and of whom the Positive and Negative Syndrome Scale (PANSS) items N1-N4, N6, G7, G13 and G16 were available (items previously identified [13]). When multiple screenings were available of the same patient, the most recent record was selected unless an older record was more complete. Data were collected in accordance with the latest version of the Declaration of Helsinki. Data were collected for diagnostic purposes, no interventions outside standard care were performed. The procedures were in accordance with local and international rules, as confirmed by the local ethical committee of the University Medical Center of Groningen, who stated that use of anonymized data from the PHAMOUS protocol for research purposes does not fall under the scope of the Medical Research Involving Human Subjects Act and therefore does not need to undergo a prior review by the medical ethical committee.

### Assessment measures

The interviews and clinician-rated scales used in this study were assessed and rated by a trained research nurse, each patient was rated by one nurse.

#### Functional outcome

Functional outcome was measured with the Health of the Nation Outcome Scales (HoNOS) [27]. The items of this clinician-rated instrument were scored on a five point scale ranging from ‘no problem’ to ‘severe to very severe problem’. The HoNOS consist of 4 subscales: behavioral problems, impairment, symptomatic and social problems. The HoNOS has shown moderately high internal consistency and moderate interrater reliability [27].

Furthermore, living situation (living in the community versus residential living) was used as a second measure of functional outcome. Patients who were living on their own, with family, friends or other housemates were characterized as ‘living in the community’, whereas patients who were living in sheltered or clinical care facilities fell into the ‘residential living’ category.

#### Symptom assessment

Symptomatology was measured with the Positive And Negative Syndrome Scale (PANSS), a commonly used semi-structured interview including three subscales, namely positive symptoms, negative symptoms and general pathology [28], on a seven point scale ranging from ranging from ‘absent’ to ‘severe’. The PANSS has shown high internal consistency and good construct validity [28].

Depressive symptoms were measured with the Calgary Depression Scale for Schizophrenia (CDSS) [29], a structured interview with nine items on a four point scale, ranging from ‘absent’ to ‘severe’. Depression as measured with the CDSS can predict outcomes differentially from negative symptoms.

#### Quality of Life

Quality of life was measured using the Manchester Short Assessment of Quality of Life (MANSA). The MANSA is a self-report questionnaire and addresses patients’ satisfaction within several psychosocial domains, including satisfaction with life as a whole, job (or sheltered employment), training/education, or unemployment/retirement), financial situation, number and quality of friendships, leisure activities, accommodation, personal safety, people that the patient lives with (or living alone), sex life, relationship with family, physical health, and mental health [30]. The twelve items that are rated on a seven point scale (‘could not be worse’ to ‘could not be better’) were used for analysis (the other four items are dichotomous (yes/no) and were excluded for methodological reasons). The MANSA has good construct validity and internal consistency [30].

### Statistical analysis

#### Confirmatory factor analysis

Based on previous work [13], the presupposed two factor structure of SA and ED was evaluated through confirmatory factor analysis (CFA) with the computer program Mplus version 7 [31]. SA (factor 1) and ED (factor 2) were entered as latent variables of the nine PANSS items. Because of violation of the multivariate normality assumption, the items were entered according to an ordinal scale using a polychoric correlation matrix. Furthermore, a robust weighted least squares estimator (WLSMV) was used, as recommended by the literature [31–34]. To measure the goodness-of-fit (GOF) of the factor structure, the following indices and cut-off criteria were used: the Comparative Fit index (CFI > .95), the Goodness-of-Fit index (GFI > .95), the Tucker-Lewis index (TLI >.95), the Root Mean Square Error of Approximation (RMSEA < 0.06), and the Weighted Root Mean Square of Residuals (WRMR < 0.90) [31]. Significantly correlated residuals were introduced into the model.

#### Regression analysis

Hierarchical multiple regression models were used to investigate the associations between SA and ED scores on the one hand (independent variables) and HoNOS, CDSS and MANSA total scores and HoNOS subscale scores on the other hand (dependent variables), while controlling for positive symptoms (total score of PANSS positive symptoms subscale), age, gender and antipsychotic medication (expressed in chlorpromazine equivalents [35]). SA (total score of PANSS items N2, N4, G16) and ED (total score of PANSS items N1, N3, N6, G5, G7 and G13) were entered in the first block, positive symptoms, age, gender, and antipsychotic medication were entered in the second block. A logistic regression model was used to examine the relationship between the negative symptom factors (independent variables) and living situation (dependent variable; 0 = non-residential, 1 = residential) controlling for the same confounders. All statistical analyses were performed with IBM SPSS Statistics version 22 (IBM Corp, Armonk, NY).

## Results

### Patient characteristics

In total, 1157 patients fulfilled the inclusion criteria. Baseline demographic and clinical characteristics are presented in Table 1. Patients were mostly male with a mean age of 45 years and they had been ill for 18,5 years on average (time since first psychotic episode). The majority of the patients were diagnosed with schizophrenia.

**Table 1:** Baseline clinical and demographic characteristics (N = 1157).

	Total sample		Non-residential		Residential		
	Mean ± SD, N (%) or median [25^th^; 75^th^ percentile] (n = 1157)	Range	Mean ± SD, N (%) or median [25^th^; 75^th^ percentile] (n = 693^a^)	Range	Mean ± SD, N (%) or median [25^th^; 75^th^ percentile] (n = 390^a^)	Range	*p*-value
**Demographics**							
Age	44.3 ± 10.8	19–72	43.6 ± 10.5	19–71	46.0 ± 11.2	21–72	< .001
Duration of illness	18.5 ± 9.7	5–55	17.21 ± 8.9	5–55	21.1 ± 10.7	5–54	< .001
Gender, % Male	775 (67.0)		435 (62.8)		295 (75.6)		< .001
*Living situation*							
Independent without partner	474 (41.0)		474 (68.4)		-		
Independent with partner	122 (10.5)		122 (17.6)		-		
With family/others	97 (8.4)		97 (14.0)		-		
Sheltered living/social pension	247 (21.3)		-		247 (63.3)		
Long-stay clinical facilities	143 (12.4)		-		143 (36.7)		
Other/unknown	74 (6.4)		-		-		
*Diagnosis*							
Schizophrenia	848 (73.3)		466 (67.2)		322 (82.6)		< .001
Schizoaffective disorder	180 (15.6)		127 (18.3)		44 (11.3)		.002
Psychotic disorder NOS	98 (8.5)		78 (11.3)		16 (4.1)		< .001
Schizophreniform disorder	18 (1.6)		15 (2.2)		3 (0.8)		.135
Delusional disorder	13 (1.1)		7 (1.0)		5 (1.3)		.765
*Psychiatric comorbidity*^b^	340 (29.4)		170 (24.5)		145 (62.8)		< .001
Substance abuse	137 (11.8)		71 (10.2)		57 (14.6)		.081
Developmental disorder	24 (2.1)		13 (1.9)		10 (2.6)		.537
Anxiety disorder	25 (2.2)		17 (2.5)		6 (1.5)		.411
Somatoform disorder	1 (0.1)		-		1 (0.3)		.360
Personality disorder	155 (13.4)		78 (11.3)		64 (16.4)		.025
Intellectual disability	37 (3.2)		15 (2.2)		19 (4.9)		.011
**Medication**							
*Antipsychotic medication*							
None	76 (6.6)		55 (8.0)		20 (5.1)		.082
Clozapine	335 (29.0)		140 (20.2)		177 (45.4)		< .001
Risperidone	194 (16.8)		76 (11.0)		37 (9.5)		.542
Olanzapine	207 (17.9)		120 (17.3)		68 (17.4)		.934
Aripiprazol	157 (13.6)		105 (15.2)		41 (10.5)		.036
Quetiapine	107 (9.2)		73 (10.5)		27 (6.9)		.039
Haloperidol	75 (6.5)		32 (4.6)		17 (4.4)		.880
Other^c^	308 (26,6)		195 (28.1)		166 (42.6)		< .001
Nr of antipsychotics	1.2 ± 0.6	0–4	1,1 ± 0.5	0–4	1.4 ± 0.7	0–4	< .001
CPZ equivalent (mg/d)^d^	350 [150; 600]	0–3037.5	300 [115; 525]	0–1800	480 [225; 750]	0–3037.5	< .001
Nr of concomitant medications	2.4 ± 2.7	0–16	1.8 ± 2.1	0–15	3.7 ± 3.1	0–16	< .001
**Outcomes**							
PANSS total	51.9 ± 15.7	30–132	48.2 ± 13.7	30–100	57.3 ± 16.7	30–132	< .001
PANSS positive	12.1 ± 4.8	7–38	11.4 ± 4.4	7–31	13.0 ± 5.3	7–38	< .001
PANSS negative	13.8 ± 6.0	7–42	12.4 ± 5.1	7–32	16.1 ± 6.5	7–42	< .001
PANSS general	25.9 ± 7.9	16–69	24.4 ± 6.8	16–50	28.1 ± 8.6	16–69	< .001
PANSS social amotivation	5 [3;8]	3–20	5 [3; 7]	3–17	6 [4; 9]	3–16	< .001
PANSS expressive deficits	9 [7;13]	6–34	8 [6;12]	6–27	11 [8; 15]	6–34	< .001
HoNOS total	9.5 ± 5.7	0–37	8.1 ± 5.1	0–26	11.7 ± 5.1	0–37	< .001
CDSS total	2.5 ± 3.1	0–17	2.5 ± 3.2	0–17	2.3 ± 2.7	0–12	.384
MANSA total	59.2 ± 12.1	14–84	59.6 ± 11.4	26–84	59.0 ± 13.2	14–84	.457

Abbreviations: CPZ: chlorpromazine; PANSS: Positive and Negative Syndrome Scale; HoNOS: Health of the Nation Outcome Scales (subtotal of items 4, 7, 8, 9 and 10); CDSS: Calgary Depression Scale for Schizophrenia; MANSA: Manchester Short Assessment of Quality of Life.

a Of 74 patients the living situation was unknown

b Nr of patients with one or more comorbid psychiatric disorder, most comorbid disorders were personality disorders (19.0%) and substance abuse disorders (16.8%).

c Other medication included: zuclopentixol (22.7%), paliperidon (13.7%), flupentixol (9.3), pimozide (7.6%), miscellaneous (46,7%).

d Chlorpromazine equivalents of antipsychotic dosage were calculated based on Gardner and colleagues [35].

### Factor analysis

In Table 2 we present the results of standardized factor loadings with significant correlated residuals. The goodness of fit indices for the CFA are good according the criteria given in the literature [36]. The RMSEA is 0.06 (CI 90%: 0.05–0.07), the WRMR is 0.86 [31], CFI is 0.99 and TLI is 0.98. All factor loadings are above 0.5.

**Table 2:** Results of confirmatory factor analysis: univariate proportions of the items and factor loadings of items N1-N4, N6, G5, G7, G13 and G16 of the PANSS (N = 1157).

		Univariate proportions of the items							
	PANSS item*	Category 1	Category 2	Category 3	Category 4	Category 5	Category 6	Category 7	Factor loading
**Factor 1 (social amotivation)**									
	N2 Emotional withdrawal	0.408	0.274	0.167	0.114	0.027	0.01	0.001	0.938
	N4 Passive/apathetic	0.367	0.243	0.208	0.088	0.072	0.02	0.001	0.872
	G16 Active social avoidance	0.593	0.207	0.135	0.035	0.024	0.004	0.002	0.674
**Factor 2 (expressive deficits)**									
	N1 Flat affect	0.361	0.22	0.199	0.118	0.095	0.003	0.005	0.821
	N3 Poor rapport	0.58	0.171	0.183	0.041	0.016	0.006	0.003	0.847
	N6 Lack of spontaneity	0.596	0.145	0.161	0.064	0.022	0.01	0.002	0.793
	G5 Mannerisms and posturing	0.707	0.144	0.124	0.014	0.004	0.004	0.003	0.504
	G7 Motor retardation	0.649	0.152	0.147	0.046	0.004	0.002	0	0.651
	G13 Avolition	0.709	0.128	0.114	0.04	0.009	0.001	0	0.585

Abbreviations: PANSS: Positive and Negative Syndrome Scale

* significant correlated residuals are (N1 with N2,N6,G7,G13,G16); (N2 with N4); (N6 with N3,G7); (G5 with G7,G16).

### Hierarchical regression

Data distributions were examined for linearity and normality. CDSS scores and HoNOS behavioral problems scores were positively skewed. The distribution was improved after applying square root transformations. Furthermore, there was no evidence for multicollinearity in the regression models. Hierarchical regression analyses were performed to investigate the relationship between both SA and ED and the outcome measures.

The analyses revealed that higher SA was significantly related to worse overall functioning (HoNOS total score), more depressive symptoms (CDSS) and worse quality of life (MANSA) (results of the final models are shown in Table 3). For the HoNOS subscales, higher SA was associated with symptomatic problems and social problems (see S1 Table). The observed associations remained significant after controlling for positive symptoms, age, gender and antipsychotic medication.

**Table 3:** Hierarchical multiple regression models for overall functioning, quality of life and depressive symptoms.

		HoNOS (N = 715) ^a^				CDSS (N = 588) ^b^				MANSA (N = 777)^c^			
Step	Variable added	β	t	p	Adj. R^2^	β	t	P	Adj. R^2^	β	t	P	Adj. R^2^
1	Social amotivation	.173	3.925	**< .001**	.158	.227	4.419	**< .001**	.066	-.184	-3.908	**< .001**	.032
	Expressive deficits	.127	2.914	**.004**		.037	.733	.464		.096	2.047	**.041**	
2	PANSS positive	.341	9.911	**< .001**	.272	.150	3.624	**< .001**	.102	-.200	-5.416	**< .001**	.086
	CPZ eq	.065	1.936	.053		-.048	-1.177	.240		.031	.866	.387	
	Age	.029	.906	.365		-.079	-1.964	**.050**		.137	3.886	**< .001**	
	Gender	.023	.699	.485		-.130	-3.236	**.001**		-.066	-1.855	.064	

Abbreviations: PANSS: Positive and Negative Syndrome Scale; CPZ: chlorpromazine; HoNOS: Health of the Nation Outcome Scales (subtotal of items 4, 7, 8, 9 and 10); CDSS: Calgary Depression Scale for Schizophrenia; MANSA: Manchester Short Assessment of Quality of Life.

^a^ Overall adjusted model *R*^2^ = .278, *F*(6.708) = 45.357, *p* < .001

^b^ Overall adjusted model *R*^2^ = .111, *F*(6.576) = 12.035, *p* < .001

^c^ Overall adjusted model *R*^2^ = .093, *F*(6.751) = 12.858, *p* < .001.

With regard to ED, higher scores were related to significantly worse overall functioning (HoNOS total score) and depressive symptoms. A positive relationship between ED and quality of life was found, indicating that higher ED was associated with higher quality of life. Higher ED scores were associated with higher scores on the HoNOS impairment subscale (cognitive and psychical or disability problems), the behavioral problems subscale and the social problems subscale. Logistic regression analyses revealed that ED was associated with residential living status (living in sheltered or clinical care facilities), which remained significant after controlling for confounders (Table 4).

**Table 4:** Logistic regression model for living situation: admission to sheltered or clinical facility (N = 1018).

Step^a^	Variables	B	OR	95% C.I. for OR
1	Social amotivation	-.053	.948	.893–1.007
	Expressive deficits	.118	1.126	1.080–1.174*
	PANSS positive	.049	1.050	1.018–1.083*
	CPZ equivalent	.001	1.001	1.001–1.002*
	Age	.019	1.019	1.006–1.033*
	Gender	-.531	.588	.428–.808*

Abbreviations: panss: Positive and Negative Syndrome Scale; cpz: chlorpromazine; OR = Odds ratio; C.I. = confidence interval

^a^ reference category: non-residential living

* p < .001.

## Discussion

In this study we established that the negative symptoms factor structure consisting of social amotivation (SA) and expressive deficits (ED) also holds in a chronic population with psychotic disorders. It thereby extends previous reports demonstrating two separate factors of negative symptoms in patients in the early phase of their psychotic illness [13] and factor analytic studies using the Scale for Assessment of Negative Symptoms (SANS) [37] or the Schedule for Deficit Syndrome (SDS) [38] (see for an overview [39]). Furthermore, the SA factor was associated with more depressive symptoms and worse quality of life, while the ED factor was most importantly related to residential living status. These relationships were not affected by positive symptoms, age, gender or antipsychotic dosage.

The replication of the dimensional structure of negative symptoms provides good support for the subdomains across the course of illness, which was not yet firmly established in chronic samples. Furthermore, the dimensional structure of the PANSS is an important addition to the factor analytic studies using the SANS and SDS, because the PANSS is widely used in clinical trials as well as in clinical practice and recognized as an appropriate tool for assessing negative symptoms [40]. As such, subdomains SA and ED can be used to assess differences in treatment response and eventually guide clinical practice in choosing a treatment strategy. There are a few notable differences in the PANSS factor analytic results compared to other instruments that should not go without mention. The main difference between the SANS studies and our results, is that PANSS avolition item (G13) loads on ED, while the SANS avolition items load on SA. PANSS ratings for avolition are merely based on observed behavior and could therefore be rated as a disturbance in willful initiation of behavior or facial expression, whereas the SANS avolition items may be rated more of a social motivational deficit [13]. Furthermore, the avolition item of the PANSS (G13) and the mannerisms and posing item (G5), which also loads on the ED factor in our study, have previously been reported as part of the disorganized factor of the PANSS. These items were nevertheless included in our analysis, because previous work showed that the factor loadings warranted inclusion in ED and that removal of these items did not improve the model fit [13]. The current factor loadings of G5 and G13 were comparable to this previous study.

The value of the distinction in subdomains is its relationship with functional and clinical outcomes [41]. Most importantly we found that higher ED was related to residential living (i.e. living in a sheltered or clinical care facility), while SA was related to more depressive symptoms and lower quality of life. Residential patients generally have a more severe course of illness and the poorest outcomes. This suggests that ED is more strongly associated with a more severe course of illness and poorer functional outcomes, contradicting evidence for SA as the key predictor of functioning [14,22]. A possible explanation for the relationship with residential living is that patients with ED seem more ‘ill’. That is, family, friends or health care workers may more often interpret SA as for example demoralization, indifference or laziness; extremes of ‘normal’ behavior. ED on the other hand, is more difficult to place within the frames of normal behavior and can seem more deviant and therefore lead to seeking help, for example in the form of admission to a residential care facility. Indeed, ED has been linked to neurocognitive deficits before [11,13,15,18] and was related to the impairment subscale of the HoNOS in this study (measuring cognition and disability) confirming higher disability and higher need for intensive (residential) care. Interestingly, these patients do not report lower quality of life than less disabled patients (given that higher ED was related to better quality of life; see also De Heer-Wunderink and colleagues [42]). The experience of a good ‘person-environment fit’ by the residential group may in part explain these findings.

Previous findings on the relationship between negative symptoms and depression have been inconsistent (i.e. some studies have reported an association [43–45], while others have not [46–48]). Our findings indeed suggest a relationship between depressive symptoms and negative symptoms. However, this relationship seems to be limited to SA. This suggests that the inconsistency in the relationship between depression and negative symptoms may (in part) be explained by the subdomain structure of negative symptoms. That is, when subjects demonstrate different scores on each subdomain (high on SA and low on ED or vice versa), their total of negative symptoms may be similar, while their relationship with depression is different as this is driven by SA.

Quality of life was significantly associated with SA. This is in line with previous work [13]. In addition, we found a relationship between SA and the subscale social problems of the HoNOS (S1 Table). Since quality of life has also been associated with social functioning [49,50], this leads us to suggest that the relationship between SA and quality of life is of an indirect nature. That is, SA causes problems with social functioning, which in turn has an effect upon the subjective quality of life. A mediation analysis demonstrated that the relationship between SA and quality of life was indeed influenced by social problems (partial mediation) (S1 Fig). However, since we did not explicitly state any hypothesis with regard to this relationship, this interpretation should be treated with caution. Higher ED was associated with better quality of life. The direction of this association is surprising and the strength of the association increased upon including positive symptoms in the regression model. This suggests that other factors influence this relationship, which makes this result difficult to interpret with the current data and deserves further investigation.

Taken together, our results seem to indicate that both factors differentially relate to distinct aspects of functioning. SA seems to be most strongly related to social-emotional aspects of functioning, reflected in associations with depressive and psychological symptoms (HoNOS subscale) and quality of life. ED on the other hand, seems to be more strongly related to aspects of everyday functioning and behavioral problems, as reflected by its associations with living situation, cognitive and disability problems (HoNOS impairment subscale) and the behavioral problems subscale of the HoNOS.

Keeping in mind that replication is needed in both chronic and other samples, some clinical implications of these findings could be cautiously suggested. Considering that SA has been linked to deficits in anticipatory pleasure [15–17], the individually oriented Cognitive Behavioral Therapy (CBT) model constructed by Staring and colleagues [51] that specifically aims at reducing negative symptoms by targeting dysfunctional beliefs including experiencing pleasure, could be particularly suited to target SA. For ED, the loss of initiative factor, personalized rehabilitation approaches aimed at examining each patient’s wishes and strengths, and accepting and working around the impairments, may be most suitable. These could include the rehabilitation approach by Anthony and colleagues [52], or compensatory strategies such as Cognitive Adaptation Training [53] or Cognitive Compensatory Training [54]. Some pharmacological treatments have shown to selectively impact SA and ED. For example, add-on mirtazapine or selegiline showed a selective effect on SA, while add-on galantamine showed specific effects on ED, and amisulpride affected both subdomains [21]. However, further research into the effects of drugs on the specific subdomains is needed.

Future research should focus on the distinction between social-emotional functioning and everyday activities to further disentangle the differential clinical correlates of both factors and to elucidate the inconsistencies in the literature. Longitudinal studies should investigate whether early interventions are useful in preventing the development of subdomain related functional problems. Intervention studies that take the subdivision of negative symptoms into account are still rare. Further, it would be useful to investigate whether the subdomains retrieved from the PANSS and SANS are interchangeable (which one would expect based on the high correlation between the PANSS negative subscale and the SANS [55]), in order to examine whether inconsistencies with regard to the functional correlates can be explained by the scale that is used. Efforts have been made in developing scales which reliably measure both subdomains of negative symptoms [56,57].

Strengths of this study are its large sample size and the fact that the data were derived from a Routine Outcome Monitoring database for which patients were not selected for research purposes and therefore are representative of the real-world population. Another strength of our study is that we did not only focus on functional outcomes but on depression and subjective quality of life as well. A limitation is the relatively low negative symptom scores (on average a rating of ‘minimal’ on each item). The lack of inclusion criteria with regard to negative symptom severity may have biased our results. Future research with patients with more profound negative symptoms is necessary to further investigate whether the relationships that we found are also applicable to those with severe negative symptoms. Furthermore, we were not able to explore proposed underlying mechanisms of SA and ED because cognitive measures and measures of anticipatory pleasure were not part of the standard PHAMOUS screening. Different neurobiological correlates have been proposed for lack of interest versus lack of initiative [58], concepts related to the present two negative factors, which deserve further investigation.

In conclusion, this study replicates the multidimensionality of negative symptoms and showed unique correlates of these two factors. Our results suggest that SA is predominantly related to social-emotional aspects of functioning, and that ED is particularly related to aspects of everyday functioning. Better understanding of the negative symptom subdomains is of value in developing treatments targeting negative symptoms in schizophrenia, which still represent an unmet need in this patient population.

## Supporting Information

S1 Data Selected variables phamous study. (SAV)

S1 Fig Standardized regression coefficients, standard errors and p-values for the relationship between social amotivation and quality of life (Fig a) as mediated by social problems (Fig b). Analysis were conducted in Mplus and corrected for age, gender and chlorpromazine equivalents.
(DOCX)

S1 Table Hierarchical multiple regression models for HoNOS subscales (results of final models are shown). (DOCX)
