# Supplementary material for: Confirmatory Factor Analysis and Differential Relationships of the Two Subdomains of Negative Symptoms in Chronically Ill Psychotic Patients
Source: PLoS One. 2016 Feb 19;11(2):e0149785. doi: 10.1371/journal.pone.0149785 (PMC4760738; doi:10.1371/journal.pone.0149785)
Supplement: S1 Fig — Standardized regression coefficients, standard errors and p-values for the relationship between social amotivation and quality of life (Fig a) as mediated by social problems (Fig b). Analysis were conducted in Mplus and corrected for age, gender and chlorpromazine equivalents. (DOCX) [file pone.0149785.s002.docx]

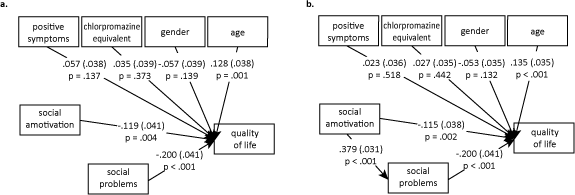


**S1 Figure**. Standardized regression coefficients, standard errors and p-values for the relationship between social amotivation and quality of life (Figure **a**) as mediated by social problems (Figure **b**). Analysis were conducted in Mplus and corrected for age, gender and chlorpromazine equivalents.
