# Supplementary material for: Confirmatory Factor Analysis and Differential Relationships of the Two Subdomains of Negative Symptoms in Chronically Ill Psychotic Patients
Source: PLoS One. 2016 Feb 19;11(2):e0149785. doi: 10.1371/journal.pone.0149785 (PMC4760738; doi:10.1371/journal.pone.0149785)
Supplement: S1 Table — (DOCX) [file pone.0149785.s003.docx]

**S1 table. Hierarchical multiple regression models for HoNOS subscales.**

|  |  | **Behavioral problems 1-3^a^** | | | | **Impairment 4-5^b^** | | | | **Symptomatic problems 6-8^c^** | | | | **Social problems 9-12^d^** | | | |
| --- | --- | --- | --- | --- | --- | --- | --- | --- | --- | --- | --- | --- | --- | --- | --- | --- | --- |
| **Step** | **Variable added** | **β** | **t** | **p** | **Adj. R^2^** | **β** | **t** | **p** | **Adj. R^2^** | **β** | **t** | **p** | **Adj. R^2^** | **β** | **t** | **p** | **Adj. R^2^** |
| 1 | Social amotivation | .013 | .268 | .789 | .050 | .021 | .434 | .665 | .053 | .211 | 4.857 | **<.001** | .111 | .154 | 3.369 | **.001** | .182 |
|  | Expressive deficits | .169 | 3.581 | **.000** |  | .170 | 3.598 | **<.001** |  | -.026 | -.602 | .547 |  | .236 | 5.251 | **<.001** |  |
| 2 | PANSS positive | .065 | 1.733 | .083 | .121 | .063 | 1.698 | .090 | .121 | .425 | 12.520 | **<.001** | .280 | .171 | 4.818 | **<.001** | .213 |
|  | CPZ eq | .071 | 1.941 | .053 |  | .067 | 1.834 | .067 |  | .059 | 1.780 | .075 |  | .069 | 2.012 | **.045** |  |
|  | Age | .245 | 6.936 | **.000** |  | .242 | 6.846 | **<.001** |  | -.033 | -1.040 | .299 |  | .012 | .371 | .711 |  |
|  | Gender | -.038 | -1.074 | .283 |  | -.035 | -.986 | .324 |  | -.022 | -.693 | .488 |  | .035 | 1.036 | .301 |  |

Abbreviations: HoNOS: Health of the Nation Outcome Scales; PANSS: Positive and Negative Syndrome Scale; CPZ eq: chlorpromazine equivalents.

^a^ Overall adjusted model *R*^2^ = .128, *F*(6.728)=17.710, *p*<.001

^b^ Overall adjusted model *R*^2^ = .128, *F*(6.728)=17.672, *p*<.001

^c^ Overall adjusted model *R*^2^ = .286, *F*(6.722)=47.816, *p*<.001

^d^ Overall adjusted model *R*^2^ = .220, *F*(6.726)=33.795, *p*<.001
